# Radial breathing mode of carbon nanotubes subjected to axial pressure

**DOI:** 10.1186/1556-276X-6-492

**Published:** 2011-08-11

**Authors:** Xiao-Wen Lei, Qing-Qing Ni, Jin-Xing Shi, Toshiaki Natsuki

**Affiliations:** 1 Interdisciplinary Graduate School of Science & Technology, Shinshu University, Ueda, Japan; 2 Key Laboratory of Advanced Textile Materials and Manufacturing Technology Ministry of Education, Zhejiang Sci-Tech University, Hangzhou 310018, P. R. China; 3 Department of Functional Machinery & Mechanics, Shinshu University, Ueda, Japan

## Abstract

In this paper, a theoretical analysis of the radial breathing mode (RBM) of carbon nanotubes (CNTs) subjected to axial pressure is presented based on an elastic continuum model. Single-walled carbon nanotubes (SWCNTs) are described as an individual elastic shell and double-walled carbon nanotubes (DWCNTs) are considered to be two shells coupled through the van der Waals force. The effects of axial pressure, wave numbers and nanotube diameter on the RBM frequency are investigated in detail. The validity of these theoretical results is confirmed through the comparison of the experiment, calculation and simulation. Our results show that the RBM frequency is linearly dependent on the axial pressure and is affected by the wave numbers. We concluded that RBM frequency can be used to characterize the axial pressure acting on both ends of a CNT.

## 1. Introduction

Radial breathing mode (RBM) of carbon nanotubes (CNTs) is a low frequency mode, but accounts for the strongest feature observed in the CNT Raman spectrum. For the RBM, all of the carbon atoms in a CNT move in the radial direction synchronously, which generates an effect similar to "breathing" [1,2]. This mode is unique to CNTs, and is not observed in other carbon systems [3]. Resonant Raman measurement of the RBM in CNTs is a standard, straightforward method for precisely determining the diameter of a CNT, distinguishing the CNT chiral-index assignments, or characterizing CNT conglomerates [4-7]. For CNTs, pressure studies are motivated by the need to investigate mechanical stability, pressure-induced phase transitions (such as vibrational characteristics), and the effects of intertube interactions. In this letter, the RBM frequency of CNTs subjected to axial pressure is studied using an elastic continuum mechanics model. Single-walled carbon nanotubes (SWCNTs) are described as an individual elastic shell and double-walled carbon nanotubes (DWCNTs) are considered as two shells coupled through the van der Waals (vdW) force interaction between them. The interaction of the vdW force between the inner and outer tubes and the effect of axial pressure are incorporated into the formulation. We consider the effects of the influences of the axial half-wave number *m*, circumference wave number *n*, nanotube diameter, and the aspect ratio *L*/*D *of the nanotubes on the RBM frequency for SWCNTs and DWCNTs exposed to varying axial pressures. Through comparison with previous results obtained from experiments and simulations, it can be seen that the continuum shell model can be used to predict the RBM frequency of CNTs exposed to various axial pressures.

## 2. Theoretical approach

### 2.1 Governing equations of SWCNTs under axial pressure

**Figure 1:**
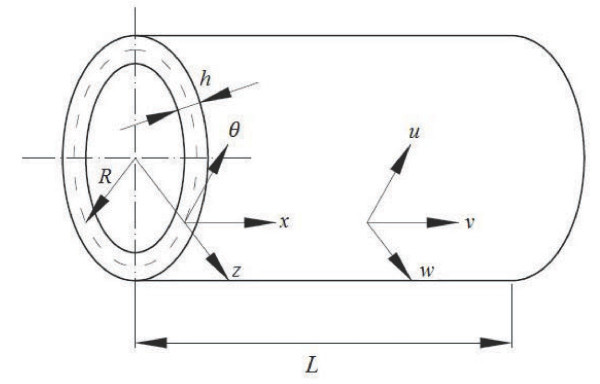
**Schematic showing the cylindrical coordinates of the CNT model used for analysis**.

A continuum elastic shell model (Figure 1) is used to analyze the characteristics of the RBM frequency of CNTs subjected to varying axial pressures. The cylindrical shell is used to designate a coordinate system (*x*, *θ*, *z*). The coordinates *x*, *θ*, and *z *refer to the axial, circumferential and radial directions, respectively. The displacements for the CNTs are *u*, *v *and *w *corresponding to the *x*, *θ*, and *z *directions, respectively. The dimensions of the nanotubes are defined as the thickness *h*, radius *R*, length *L*, Poisson's ratio *v *and density *ρ*.

**Figure 2:**
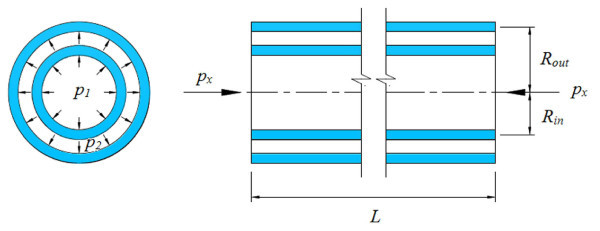
**Schematic of the DWCNT model subjected to axial pressure for analysis**. The left image displays the latitudinal cross-section, which shows the vdW force between the inner and outer tubes. The right image displays the longitudinal cross-section, which shows the axial pressure acting on both ends of the DWCNT.

Based on our previous work [8] and Love's first approximation shell theory [9], the equation of motion for simple supported CNTs is given by:(1)(2)(3)

where *Φ *= (1-*v*^2^)/*Eh *and *β *= *h*^2^/12*R*^2^; *p_x _*is the axial pressure acting on the both ends of the CNT; and *p *is the vdW interaction pressure between inner tube and outer tube in a DWCNT, which is shown schematically in Figure 2.

### 2.2 van der Waals interaction forces

To study the vibrational behavior of DWCNTs, a double-elastic shell model was developed that assumes each of the nested tubes in a CNT is an individual elastic shell, and the adjacent tubes are coupled to each other by normal vdW interactions. The pressures exerted on the inner and outer nanotubes through the vdW interaction forces (Figure 2) are given as(4)(5)

*w_k _*(*k *= 1, 2) are the radial displacements of the inner and outer nanotubes, and *c_ij _*(*i*,*j *= 1, 2) is the vdW interaction coefficient between nanotubes, which can be estimated using the Lennard-Jones potential [10]:(6)

Where(7)(8)

where *a *is the carbon-carbon bond length (0.142 nm); *R_i _*and *R_j _*are the inner and outer radii of the DWCNTs; and *σ *and *ε *are the vdW radius and the well depth of the Lennard-Jones potential, respectively. The vdW parameters of the DWCNTs in the Lennard-Jones potential are *ε *= 2.967 meV and *σ *= 0.34 nm (from Saito *et al*. [11]).

### 2.3 RBM frequency of DWCNTs

To model the vdW force, we substitute Eqs. (4) and (5) into Eqs. (1)-(3). The governing equations for the RBM frequency of inner and outer tubes of DWCNTs subjected to an axial pressure can be expressed as:

For inner tube(9)(10)(11)

For outer tube(12)(13)(14)

where *r *and *R *are the radius of inner tube and outer tube of the DWCNT, respectively. To simplify the calculation, Eqs. (9)-(14) can be rewritten as(15)

where *L_ij _*are the differential operators given as:(16)

The general solution for the displacements *u_k_*, *v_k _*and *w_k _*in the inner and outer tubes of a DWCNT can be given by(17)(18)(19)

where *A_k_*, *B_k _*and *C_k _*are the longitudinal, circumferential and radial amplitudes of the displacements in the inner (*k *= 1) and outer tubes (*k *= 2), respectively. *L *is the length of CNT which is shown in Figure 1. The wave numbers *m *and *n *are the axial half-wave and circumferential numbers, respectively.

## 3. Numerical results and discussion

For the present analysis, an individual SWCNT was assumed to be a graphene sheet rolled into a cylinder and the DWCNT is considered to be two layered nanotube shells coupled by vdW interactions. The value of the thickness of sheet is 0.34 nm; the elastic modulus is 1.0 TPa; the Poisson's ratio is 0.27; the mass density of the CNTs is 2.3 g/cm^3^; and the inner and outer diameters of the DWCNTs are 2.2 nm and 3.0 nm, respectively [12].

Based on our proposed theoretical approach, we first calculate the RBM frequency of an isolated SWCNT subjected to zero pressure varying with radius. Note that the commonly used unit for the frequency of the RBM *f *is in cm^-1 ^for Raman spectroscopy experiments. However, the unit Hertz (Hz) for *ω *has been adopted for convenience in this study. Our results show that when the diameter is increased from 1.43 to 1.59 nm, the RBM frequency of a SWCNT monotonically decreases from 33.03 to 26.76 THz. To extract the correct parameters for the elastic continuum model, we compared the present work with other approaches. According to Raman scattering technique by Jorio *et al*. [13], the frequency of a RBM ranges from 33.18 to 28.46 THz for SWCNTs with diameters of 1.43 to 1.65 nm when structural factors are considered. Furthermore, Peica *et al*. [6] used tip-enhanced Raman spectroscopy (TERS) to show that with a change in diameter from 1.35 to 1.63 nm, the RBM frequency changes from 33.44 to 28.29 THz for SWCNTs. Kurti *et al*. [14] used first-principles calculations to show that the frequency of the RBMs were between 32.72 and 28.14 THz for SWCNTs with diameters ranging from 1.35 to 1.56 nm. Batra *et al*. [3] also investigated the RBM frequency of CNTs using the molecular dynamics (MD) method and finite element method (FEM). They found that the RBM frequency ranged from 31.55 (31.22 for FEM) to 27.40 (27.26) THz for SWCNTs with diameters between 1.35 to 1.56 nm. Our calculated frequencies of the RBM of SWCNTs with varying diameters not exposed to axial pressure agree closely with these previously reported values. This verifies that the continuum elastic shell model can accurately describe the RBM frequency of CNTs.

**Figure 3:**
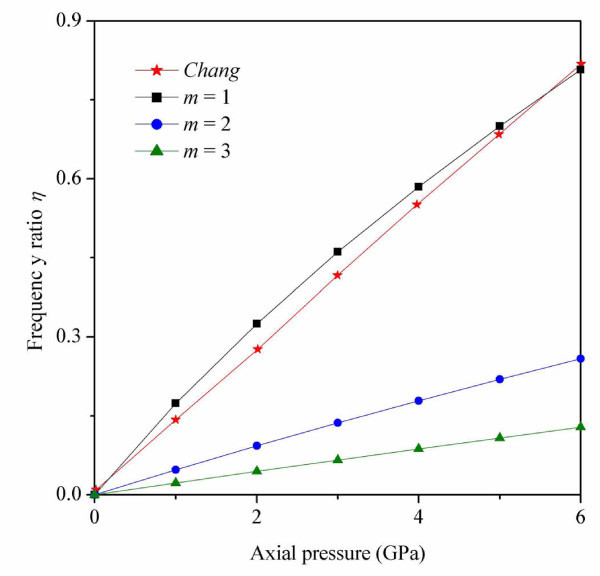
**RBM frequency ratios for SWCNTs with axial half-wave numbers (*m*) varying from 1 to 4**. The results from the nonlinear stick-spiral model by Chang *et al*. [15] are also presented for comparison.

The RBM frequency ratios of SWCNTs with different axial half-wave numbers subjected to axial pressure in shown in Figure 3. The frequency ratio *η *is defined as:(20)

where *f_p _*is the frequency of the RBM with exposed to axial pressure, and *f_0 _*is the frequency of RBM not exposed to axial pressure. Note that the RBM frequency ratio *η *is sublinear with respect to increasing axial pressure. We also found that the axial half-wave number *m *plays a critical role in this increasing frequency ratio. When the axial half-wave number increases, the increment of change in the speed of the frequency ratio becomes much smaller. The results from nonlinear stick-spiral model, presented by Chang *et al*. [15], are also shown for comparison. The models show good agreement when the axial half-wave number *m *is 1. These results confirm that the largest contribution to the RBM for a SWCNT comes when *m *= 1. By contrast, *m *≥ 2 wave numbers are in the minority.

**Figure 4:**
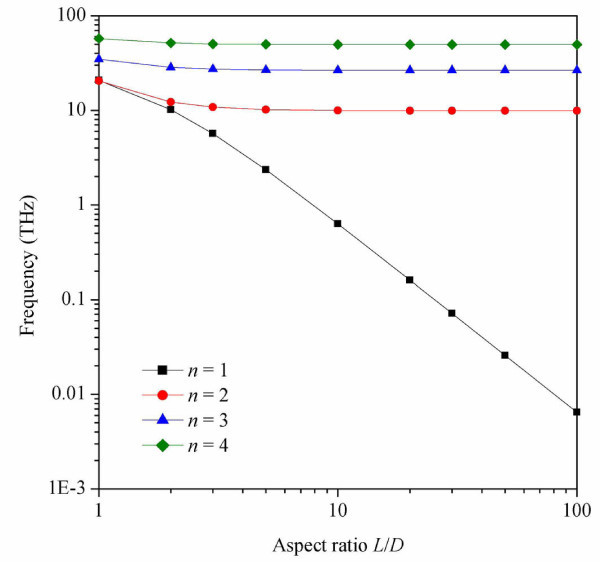
**RBM frequencies of the SWCNTs as a function of aspect ratio**. The SWCNTs have the same axial half-wave numbers, *m *= 1, and axial pressures, *p_x _*= 1 TPa. However, they have circumference wave numbers *n *that vary from 1 to 4.

Figure 4 shows the RBM frequencies of SWCNTs as a function of the aspect ratio *L*/*D *(*D *is the diameter of the SWCNT). The frequencies of the SWCNTs grow sublinear logarithmically with an increasing aspect ratio. When the circumferential wave *n *is 1, the frequency of the RBM decreases dramatically with increasing aspect ratio. This is especially true for larger aspect ratios, and the variational trend of the RBM frequency is quite different for *n *= 2, 3, 4 or higher. This is caused by the misalignment of the tubes for the circumferential wave *n *= 1, which makes the entire SWCNTs unstable. The unstable SWCNTs with *n *= 1 can be affected by external factors more easily than those with other circumferential waves numbers. This phenomena is unique to SWCNTs with *n *= 1, and cannot be observed with other circumferential waves numbers. However, the RBM frequency is largely unaffected by the aspect ratio of the tube when *n *≥ 2.

**Figure 5:**
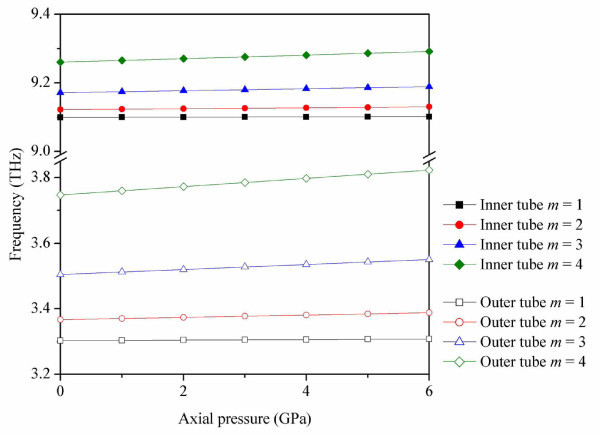
**RBM frequencies of DWCNTs as a function of axial pressure**. With axial half-wave numbers (*m*) that vary from 1 to 4.

In the following, an analysis of the RBM frequency of inner and outer tubes in DWCNTs subject to axial pressure is carried out. Figure 5 shows the RBM frequencies of DWCNTs with different axial half-wave numbers of *m *= 1-4 as a function of increasing axial pressure for inner and outer tubes. The wave number in the axial direction plays an important role in determining the RBM frequency of DWCNTs. Thus, the larger mode numbers result in higher RBM frequencies with increasing axial pressure. The RBM frequencies of both inner and outer tubes have positive linear relationships with pressure acting on both ends of DWCNTs. Compared with RBM frequencies of inner tubes, the frequencies of outer tubes are more sensitive to the varying wave numbers and axial pressure shown in Figure 5. The frequencies of inner and outer tubes vary over different ranges when the DWCNTs are subjected to the same axial pressures. For a given interval of axial pressure, the RBM frequency of outer tubes is a little higher than that of inner tubes. The reason is that the RBM frequency with smaller radius CNT is much less than that of longer radius CNT, which has been mentioned in the beginning.

## 4. Conclusions

Based on elastic continuum mechanics, we studied the RBM frequency of simply-supported CNTs exposed to axial pressure. The SWCNTs were modeled as individual elastic shells, and the DWCNTs were considered to be two layered nanotube shells coupled by vdW interactions. The effects of the wave numbers, aspect ratio and axial pressure are discussed in detail. It can be seen through comparison with previous experimental and simulation investigations on the RBM frequency of isolated SWCNTs with increasing radius and the RBM frequency ratio with increasing pressure, the continuum shell model can be used to predict the RBM frequency of CNTs subject to an axial pressure. The results of the CNTs exposed to an axial pressure show that the RBM vibration frequency is sensitive to both the vibrational mode and axial pressure, while the frequency of the RBM is hardly affected by the aspect ratio. We are now processing the theoretical analysis on vibrational properties of SWCNTs and DWCNTs subjected to axial pressure in order to provide further quantitative and qualitative experiments and simulations on RBM of CNTs.

## Abbreviations

RBM: radial breathing mode; DWCNT: double-walled carbon nanotube; CNT: carbon nanotube; vdW: van der Waals; SWCNT: single-walled carbon nanotube; Hz: Hertz; TERS: tip-enhanced Raman spectroscopy; MD: molecular dynamics; FEM: finite element method.

## Competing interests

The authors declare that they have no competing interests.

## Authors' contributions

The work presented here was carried out in collaboration between all authors. XWL carried out the theoretical analysis and wrote the paper. QQN discussed the results and helped write the manuscript. JXS verified the method and created some of the figures. TN helped define the research theme, design the methods. All authors read and approved the final manuscript.
